# Developing medical professionalism in future doctors: a systematic review

**DOI:** 10.5116/ijme.4bda.ca2a

**Published:** 2010-05-14

**Authors:** Vimmi Passi, Manjo Doug, Ed Peile, Jill Thistlethwaite, Neil Johnson

**Affiliations:** Warwick Medical School, University of Warwick, UK

**Keywords:** Keywords: Professionalism, medical students, curriculum, teaching, assessment, role modelling

## Abstract

Objectives: There are currently no guidelines on the most effective ways of supporting medical students to develop high standards of medical professionalism. The aim of this review is to summarise the evidence currently available on methods used by medical schools to promote medical professionalism.

Methods: We performed a systematic search of electronic databases (Medline, PsychInfo, British Education Index, Educational Resources Information Centre, Sociological Abstracts and Topics in Medical Education) from January 1998 to October 2008. Outcomes studied were methods used to support and promote the development of professionalism in medical students.

Results: We identified 134 papers and five main themes for supporting the development of professionalism in medical students: curriculum design, student selection, teaching and learning methods, role modelling and assessment methods. However, the level of empirical evidence supporting each of these methods is limited.

Conclusions: Identification of these five areas helps medical schools to focus the emphasis of their approaches to developing professionalism and identifies future research areas. This review offers a preliminary guide to future discovery and progress in the area of medical professionalism.

## Introduction

Medical professionalism forms the basis of the contract between doctors and society and it is thus imperative that professionalism is incorporated into the undergraduate curriculum.^1^^, ^^2^ The importance of teaching medical professionalism has been emphasised in many countries worldwide. However, at present there are no guidelines on how professionalism should be developed in medical students. A Lancet review in 2001 emphasised the importance of a commitment to the teaching of professionalism to medical students and suggested that rigorous research was required in this area.3 Although recent literature has highlighted the continuing challenges of educating for medical professionalism over the past seven years; the literature on professionalism has grown exponentially. This expansion in the literature, taken together with the priority that the teaching and assessment of professionalism has assumed for medical schools worldwide, means that we can begin to make recommendations on the most effective methods for medical schools. The aim of this systematic review is to evaluate the evidence supporting the development of professionalism in medical students. A summary of the key methods used to support the development of professionalism is presented. However, first it is necessary to review briefly the key definitions of medical professionalism in use, as the clear specification of the curriculum is highly dependent on these.

### Defining medical professionalism

There are numerous definitions of medical professionalism provided by major medical organisations. International experts in North America and Europe have been instrumental in defining professionalism. The General Medical Council’s publication Good Medical Practice describes the duties of a doctor as ‘providing good clinical care, maintaining good medical practice, teaching and training, relationships with patients, working with colleagues, probity and health’.4 In the UK, the General Medical Council guidance to medical schools on change in the undergraduate curriculum, *Tomorrow’s Doctors,* has been influential to the teaching of professionalism emphasising the attitudes and behaviour.^5^

 The American Board of Internal Medicine (ABIM) commissioned *Project Professionalism*, which sought to define the components of medical professionalism.^6^ Professionalism as defined by the ABIM, has six components: altruism, accountability, excellence, duty, honour/integrity and respect. Similarly, *CANMEDS* a project of the Royal College of Physicians and Surgeons of Canada, delineated a competency framework and specifies seven roles expected of the competent specialist: medical expert, communicator, collaborator, manager, health advocate, scholar and profesional.^7^ In 2002, a combined North American and European Internal Medicine Boards project published the *Physician’s Charter* – a declaration on medical professionalism requirements for the new millennium.^8^ It has subsequently been endorsed by over 120 medical organisations and translated into ten languages. The *Physician’s Charter* consists of three fundamental principles (patient welfare; patient autonomy and social justice) and a set of ten professional responsibilities (commitment to competence, honesty, confidentiality, relationships, quality of care, access to care, distribution of finite resources, scientific knowledge, managing conflicts, responsibilities).^8^ Most recently, the Royal College of Physicians of London’s Working Party on Medical Professionalism has defined medical professionalism succinctly as ‘a set of values, behaviours and relationships that underpin the trust the public has in doctors, with doctors being committed to integrity, compassion, altruism, continuous improvement, excellence and teamwork’.^9^ It is however, important to emphasize that there still remains uncertainty about what professionalism actually is and although medical educators primarily frame professionalism as a list of characteristics or behaviours, many sociologists favour theories that incorporate political, economic and social dimensions into the understanding of the nature and function of professionalism.^10^ In addition, moralists will argue that professionalism should be seen clearly as an aspect of personal identity and character which develops over time.^11^ Finally, many authors have moved the focus from the individual to the institution and have argued that professionalism should be inculcated within medical schools with complete integration of a culture of professionalism involving staff, faculty, residents and students.^12^^-^^14^

## Methods

### Search strategy and selection criteria

We systematically searched the following six electronic databases: Medline; PsychINFO; EMBASE; Educational Resources Information Centre (ERIC); Sociological Abstracts; and Topics in Medical Education (TIMELIT) from 1998 to October 2008. Only those articles that had abstracts available in English were included and we limited the retrieved articles to the past ten years on the basis that a major review on this subject had been published in 2001 and we wished to focus particularly on progress in this field since that time.^3^ Medical subject headings (MESH) and key words included were: “medical education;” combinations of “professionalism;” “professional behaviour/practice;” “medical students;” and “undergraduate medical students” (Table 1).

**Table 1:** The inclusion and exclusion criteria for the systematic review

	Inclusion Criteria	Exclusion Criteria
Professionalism	Defined by the RCP as:	Non-medical students
	Medicine is a vocation in which a doctor's knowledge, clinical skills and judgment are put in the service of protecting and restoring human well-being. This purpose is realised through a partnership between patient and doctor, one based on mutual respect, individual responsibility and appropriate accountability. Doctors are committed to:	Non-medical teachers/doctors

	Integrity	
	Compassion	
	Altruism	
	Continuous improvement	
	Excellence	
	Working in partnership with members of the wider healthcare team	
	These values, which underpin the science and practice of medicine, form the basis for a moral contract between the medical profession and society.	
Medical Education	Additional components of medical education focused on to promote professionalism:	
	Student selection/admissions to medical schools	
	Curriculum design and competency based curriculum	
	Teaching	
	Role modelling	
	Hidden curriculum	
	Assessment	
Medical students	Undergraduate medical students	Postgraduate students
		Doctors
		Residents
Design	Systematic reviews	Letters
	Reviews	Editorials
	Original research	
Language	English	Non English
Publication date	Papers published from 1998 - October 2008	Papers published before 1998

To ensure that all key studies were included we also searched the reference lists of studies identified through the primary search, and conducted a secondary search using themes emerging from studies identified by the primary search. A Reference Manager (version 11) library was created to catalogue these references. The bibliography created from the search produced a list of references with title and/or abstract. The eligibility of each study was assessed jointly by two investigators (VP, MD), based on our inclusion criteria. Studies were included in the review if they were about ‘medical professionalism’ and included ‘undergraduate medical students.’ Studies were excluded if they reported professionalism in residents or postgraduate students. We also excluded studies that were not published as full reports, such as conference abstracts/proceedings, letters and editorials. If a clear decision regarding inclusion could not be made from reading the abstract, the full text article was obtained. Our main outcomes were methods used to support and promote the development of professionalism in undergraduate medical students.

### Data extraction

Two assessors independently data extracted full text studies (VP, MD) and the decision to include each paper was made based on the inclusion criteria. The extracted information was systematically collated onto data extraction forms.

### Analysis

We used SPSS version 15 to organise, manage and analyse the search findings. Frequency tables were used to summarise the studies’ characteristics. Details of how professionalism is integrated and developed in undergraduate medical students were summarised in detail, although any statistical integration of the data findings was not possible due to the predominantly descriptive nature of the designs. The findings were integrated in a narrative structure.

## Results

**Figure 1:**
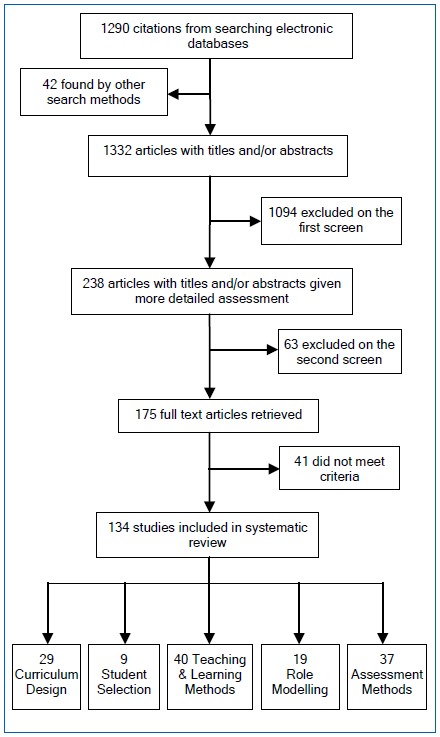
Flow diagram of the search process

The search strategy identified 1332 citations, of which 238 were potentially relevant. After further screening of the 238 titles and/or abstracts, 175 full text articles were obtained following which 41 were excluded as they did not meet the inclusion criteria leaving 134 papers for detailed review (Figure 1).

### Focus of included studies

Five main areas for supporting the development of professionalism in medical students emerged from the analysis of the papers: curriculum design; student selection; teaching and learning methods; role modelling and assessment methods (Table 2). Each of the five areas is described in detail below. These five areas are in accordance with those described by other authors.^13^^, ^^15^ In all five areas most of the studies are descriptive. The area with the greatest proportion of analytical studies is the assessment of professionalism.

**Table 2:** Summary of the main focus of articles and associated references identified by the included studies

Main focus of articles	References
Curriculum Design	2, 3, 13-39
Student Selection	40-48
Teaching and Learning Methods	49-87
Role Modelling	88-106
Assessment	107-143

### Curriculum design

The systematic review identified 29 papers focusing on curriculum design (see Table 2). Medical schools throughout the world have acknowledged the importance of incorporating professionalism into the undergraduate medical curriculum and there are many descriptions of various curricula that medical schools have designed to integrate professionalism.^18^^,^^24^^-^^25^^,^^29^^-^^34^^,^^38^^-^^39^

However, there is currently no commonly accepted theoretical model being used for the integration of professionalism into the undergraduate curriculum and further analytical research is required to compare the approaches between the different medical schools. The one consistent theme emerging from the literature on curriculum design is that academic institutions must accept greater responsibility and accountability for the professionalism of future doctors.^15^ In the UK, the curriculum must be designed, delivered and assessed to ensure that graduates demonstrate all the outcomes specified in Tomorrow’s Doctors.^5^

### Student selection

This systematic review highlighted nine papers focusing on student selection (Table 2). Some medical schools try to avoid selecting students who have unprofessional personal characteristics likely to affect their ability to care for patients and work within a team.^40-^^41^ There is a correlation between unsatisfactory behaviours on record at medical school and subsequent disciplinary action.^42^ It is argued that to reduce the risk of graduates behaving unprofessionally, potential entrants should be assessed for non cognitive attributes that may be predictive of future professional behaviours in addition to their prior academic achievement.^43^ However, the understanding of which attributes might be predictive remains very limited and ( partly as a consequence) there are as yet no tools with demonstrable validity and reliability for selection on the basis of the likelihood of being able to develop professionalism. Although there is currently no evidence to support the adoption of any single selection process, a number of models are emerging that have been designed to consider potentially relevant character traits. Examples include the use of the *Personal Qualities Assessment* tool or the *Multiple Mini Interviews (MMI)* to test for relevant attributes; the assessment of candidate suitability for a problem based curriculum, a self reported measure of dysfunctional personality characteristics that inhibit the development of working relationships with others and measures of moral orientation prior to the making of a moral decision.^7^^, 40, ^^44^^-^^48^

### Teaching and learning methods

This systematic review found forty papers focusing on teaching and learning methods (Table 2).

The papers were mainly descriptive, each describing one method with very little comparison and effective evaluation of the different teaching methods. Table 3 summarises the key teaching methods identified in the literature. Three prominent themes in the teaching and learning of professionalism emerged. Firstly, there was the development of patient centred approaches to care.^55^^, ^^57^^, ^^80^ Secondly, there was the focus on encouraging the development of reflective practice.^50^^, ^^52^^, ^^58^^, ^^64^^, ^^67^^, ^^80^^-^^81^^, ^^83^ The third was the development of ethical approaches to practice. ^49^^,^^51^^,^^53^^,^^55^^,^^73^^,^^81^ As the teaching of professionalism is highly context dependent and evolutionary, the teaching and learning methods identified are very helpful in planning varied learning outcomes throughout the undergraduate curriculum. Medical educators may choose to use a variety of methods throughout the curriculum.

**Table 3:** Teaching and learning methods

Teaching Method	References
Experiential; Reflective Practice	50,51,53,56,62-64,69,71,77,79,83,86
Clinical Contact including tutor feedback	55-57,65,80
Undergraduate Ethics Teaching	49,51,54,55,61-62,67,73,78,81
Problem Based Learning	59,74,76,84,85
Role Play Exercises	56,138
Bedside Teaching	53,63,72,75
Educational Portfolios	62,69
Videotaped Consultation Analysis	52,58
Significant Event Analysis	58,60,64,83
Workshops; Interactive Lectures	50,138
Humanities writing: reading poetry/prose Related to patient and doctors	81
Mentoring Programmes	85

### The influence of role modelling

This systematic review highlighted 19 papers focusing on role modelling as shown in Table 2. Role models are undoubtedly important in professional character formation.^88^ Role modelling is an important method to impart the values of the medical profession, as excellent role models will always encourage professional behaviour in future doctors.^89^ Medical educational programmes should use positive role modelling as an effective teaching tool.^90^ Role modelling takes place in three interrelated educational environments which are the formal, informal and hidden curriculum. The informal curriculum is defined as an ‘unspecified, predominantly ad hoc and highly interpersonal form of teaching and learning that takes place among and between faculty and students.^91’^ The hidden curriculum has been defined as a ‘set of influences that function at the level of organisational and culture’.^91^ Institutions need to involve faculty members in an analysis of their educational environment and assist them in adapting their teaching and role modelling.^92^^-^^97^ Medical educators need to ensure that learners can understand and reflect on negative role modelling and use it as an effective learning experience.^98^^-^^100^ Three characteristics of good role models have recently been described: clinical competence, teaching skills and personal qualities, and this provides a potentially promising framework for the analysis and continuing development of role models.^90^

### Assessment of professionalism

This review found 37 papers focusing on the assessment of medical professionalism (Table 2).

The different methods of assessment are summarised in Table 4. Several major review articles regarding the assessment of professionalism have recently been published.^110^^,^^120^^,^^124^^,^^126^^,^^143^ Arnold, (2002) reviewed over 170 papers and classified assessment instruments into three groups namely those addressing professionalism as part of general clinical competence, those approaching professionalism as a single construct and those addressing separate elements of professionalism such as humanism.^110^ The author concluded that the development of good qualitative methods is needed to strengthen quantitative approaches and that further research is required regarding the environment in which assessment should take place.

**Table 4:** Methods of assessing medical professionalism

Assessment Method	References
Peer Assessment	108,109,113,116,120,125,134,136,139
Direct Observation	113, 114,123,128,135
Patient Evaluations	113,119,120,126
Objective Structured Clinical Examination	113,120,127
Standardised Patient Assessments	120,142,139
Student Evaluation Forms	122,123,131
Self Assessment	113,119,133
Educational Portfolios	117,121
Teamwork Exercises	120,139
Professionalism Mini Evaluation Exercise P-MEX	115
Attendance Records	113
Videotape Analysis	120
Single Best Answer Multiple Choice Situational Judgement Test	137

Epstein and Hundert, (2002) reviewed 195 papers, identifying a wide range of assessment tools.^120^ They found that few of the reported assessments observed students in real life situations, incorporated the perspectives of peers and patients or used measures that predict clinical outcomes. Lynch and colleagues, (2004) reviewed 191 articles published between 1982 and 2002, reporting the use of 88 instruments.^126^ The authors suggested that professionalism assessments may be organised into *content area addressed* (i.e. ethics, personal characteristics, comprehensive professionalism and diversity) and *type of outcome examined* (affective, cognitive, behavioural and environmental). The authors concluded that before developing new instruments the existing ones should be improved as many have undergone little testing particularly in terms of their predictive validity. Veloski and colleagues, (2005) conducted a systematic review of papers published between 1984 and 2002 using a panel of 12 national experts in the USA, and identified a total of 134 empirical studies.^143^ The majority of studies looked at specific components of professionalism (mainly ethical or moral reasoning), with many instruments assessing the learning environment or group behaviour rather than individual behaviour and only a few addressing professionalism as a comprehensive construct. The authors concluded that there were few well documented methods that can be used to measure professionalism formatively or summatively. Jha et al (2007) performed a systematic review of 97 studies that considered approaches to assessing and facilitating attitudes towards professionalism in medicine.^124^ Whilst recognising that the evidence in this field is limited the authors concluded that there is a need to measure global attitudes rather than attitudes towards specific issues in professionalism and a need to track attitudes throughout the curriculum. The authors demonstrated that there is no single preferred assessment method and also indicated that one reason for this is the lack of a consensus view on exactly what elements of professionalism should be assessed. In summary, although a large number of instruments have been described, the major studies suggest that there is no single instrument for measuring all aspects of professionalism.^110^^, ^^120^^,^^124^^,^^126^^,^^143^ Many authors have described the importance of a multidimensional approach with a range of instruments being used throughout the curriculum for both formative and summative assessment purposes. Workplace Based Assessment is thus important, allowing the systematic assessment of professionalism using many different assessors in different clinical settings.

## Discussion

The development of medical professionalism in undergraduate medical education serves an important societal purpose.^21^ To our knowledge, this is the first systematic review to summarise the evidence currently available on methods used by medical schools to promote the development of medical professionalism in their students. The principal strength of this study was the detailed search strategy designed to cover all approaches to the development of professionalism. The clear categorisation of the literature into five main areas will be a useful resource for medical educators, allowing institutions to draw their own conclusions and stimulate further research in the field of medical professionalism. This review does have some limitations. In particular, whilst we believe that it represents all studies published in this area during the time period concerned, given that professionalism as a concept is still relatively poorly defined we recognise that there is a chance that some keywords may have been missed during the search and thereby some papers not identified, although we believe that this is relatively unlikely given the multifaceted approach taken to the identification of the relevant literature. We also recognise limitations imposed by restricting the inclusion solely to studies reported in English; whilst we do not believe that this is likely to have resulted in significant numbers of empirical studies being missed (as the vast majority of empirical studies are published in English), we do recognise that more descriptive papers may have been missed and that this may have excluded alternative constructs of medical professionalism (particularly non Western ones). This review highlights three main challenges to supporting future doctors to develop their professionalism. Firstly, professionalism is a multifaceted concept and the lack of a consensus definition of professionalism presents a challenge to curriculum design. Secondly, there are no evidence based strategies for the teaching and assessment of professionalism and it will take time to develop and research the main areas identified in this systematic review. Thirdly, individual, societal and political expectations are continually evolving placing increasing demands on doctors. These are big challenges – we hope that this review can precipitate an open debate on the most appropriate approaches to be taken in the development of medical professionalism in future doctors.

### Selection

Selection of students presents a major challenge to medical schools.^45^^, ^^48^ There is a requirement to ensure that successful applicants will ultimately be fit to practice which includes their demonstration of professionalism.^42^ However, this review has highlighted the paucity of instruments demonstrated to predict, at the point of selection, students’ capacity to develop professionalism. Given the investment involved in undergraduate medical education, further research in this area is required. The literature suggests that perhaps the emphasis should be on de-selection rather than selection – identifying methods that can select out reliably those applicants who are unlikely to be able to develop all aspects of professionalism.

### Teaching and learning

**Figure 2:**
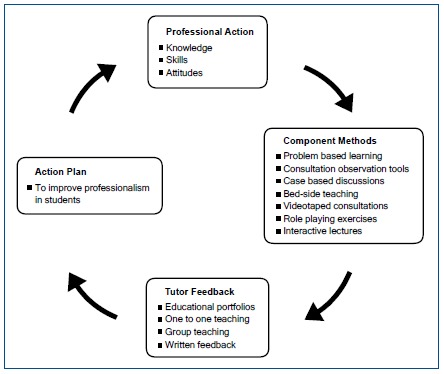
The teaching and learning professionalism cycle

This review highlights the very wide range of approaches that are available for teaching and learning professionalism. However, the very limited comparative data mean that, as yet it is not possible to identify any particular methods demonstrated to be more effective than others. Nevertheless, as professionalism is a complex construct, the evidence suggests the use of a variety of teaching methods throughout the curriculum. It is clear that the development of professionalism evolves over time by a process of exploration and reflection. Hence, a major objective of medical education should be to provide multiple learning opportunities for gaining experience in and reflecting on the concepts and principles of medical professionalism. We therefore propose that particular emphasis should be placed on learning in the clinical setting, drawing on real day to day examples. Figure 2 demonstrates how this might be done using our ‘Professionalism Cycle,’ which emphasises the importance of reflection on actions by medical students followed by feedback from the clinical teachers who will also highlight the important principles of professional practice (Figure 2). The documentation of such learning could be presented in an educational portfolio, which documents the development of professional achievements, personal reflections and learning needs throughout the curriculum.^117^^, ^^121^ The Professionalism Cycle thus demonstrates the foundations for lifelong learning and continuing professional development that is required by all doctors.

### Role modelling

In medical education, clinical teachers consciously or unconsciously act as role models for students.^92^^, ^^93^ Doctors who protect time to facilitate feedback, reflection and make a conscious effort to articulate what they are modelling in addition to providing good clinical care are most likely to be recognised as excellent role models.^89^ It is important that medical institutions consider methods to develop the professionalism of staff who act as role models.^103^^-^^104^ Personal and professional development of medical students is more likely to occur in a supportive learning environment. Hence, it is important that all medical educators to analyse their learning environments and then develop strategies to ensure that all clinical teachers demonstrate the values, attitudes and behaviours that characterise modern medical professionalism.^92^ The professional attributes associated with effective role models represent behaviours that can be modified and therefore there is a need for medical institutions to support and develop effective doctor role models.^93^^-^^97^

### Assessment

Professionalism is developmental and highly context specific, that is, professional development evolves continually throughout the curriculum and its demonstration is highly dependent on the context in which the students find themselves.^139^ The literature emphasises the importance of workplace based assessment methods using different observers marking the medical students’ professional performance. The assessment of professionalism should involve both formative and summative methods and this requires faculty input to develop effective clinical assessors. The educational portfolio has been highlighted as a potentially important tool that allows links to be created between the processes of learning and assessment and can assess the more subtle components of professionalism such as integrity, honesty and attitudes.^117^^, ^^121^ Medical schools internationally could collaborate, as there is a need to develop valid, reliable and practical programmes for the assessment of medical professionalism. In addition, it is important that medical educators are encouraged to simultaneously address the importance of monitoring of unprofessional or disruptive behaviours.^128^^, ^^131^ Consideration should be given to developing a framework for understanding approaches to and processes for dealing with unprofessional behaviour. It is important to develop institutional policies to detect unprofessional behaviours and to also develop procedures for the remediation of medical students. In summary, professionalism in medicine is undoubtedly an extraordinarily complex phenomenon. This review offers a preliminary guide to future discovery and progress in the area of medical professionalism. It is imperative that medical educators worldwide collaborate and share ideas regarding the development of medical professionalism, as we have a duty to ensure that future doctors provide high professional standards of care to our patients.
